# Efficacy and safety of Yi Shen Fang granules in elderly people with MCI: study protocol for a multicentre, randomized, double-blind, parallel-group, controlled trial

**DOI:** 10.1186/s12906-023-03940-x

**Published:** 2023-04-03

**Authors:** Zhongwei Sha, Zhenghao Zhao, Nana Li, Shuyun Xiao, Ou Li, Jie Zhang, Zhimin Li, Jian Xu

**Affiliations:** Department of Mental Diseases, Shanghai Municipal Hospital of Traditional Chinese Medicine, Shanghai University of Traditional Chinese Medicine, Shanghai, China

**Keywords:** Traditional Chinese medicine, Cognitive, Mild cognitive impairment, Clinical trial

## Abstract

**Background:**

Mild cognitive impairment (MCI) is a transitional state between normal ageing and dementia. Most MCI patients will progress to dementia within 5 years; therefore, early intervention for MCI is important for delaying the occurrence and progression of dementia. Yi Shen Fang (YSF) granules are a promising traditional Chinese medicine (TCM) treatment that shows great neuroprotective potential against cognitive impairment, as evidenced in clinical and basic studies. This trial aims to systematically evaluate the efficacy and safety of YSF granules in elderly people with MCI.

**Methods:**

This study is a multicentre, randomized, double-blind, parallel-group, controlled trial. Based on the results of previous clinical trials, 280 elderly patients with MCI will be randomly divided into a treatment group (*n* = 140) and control group (*n* = 140). The study will last 33 weeks, including 1 week of screening, 8 weeks of intervention, and 24 weeks of follow-up. The primary outcomes will be the changes in Montreal Cognitive Assessment (MoCA) and Memory and Executive Screening (MES) scores before and after the intervention. The secondary outcome measures will be homocysteine (HCY) levels, Functional Assessment Questionnaire (FAQ) scores and event-related potential (ERP) detection in typical cases. The TCM symptom scale is a combined measure of syndrome differentiation and treatment. During this study, the classifications and characteristics of adverse events, the times of occurrence and disappearance, the measures of treatment, their impact on the primary disease, and outcomes will be reported truthfully.

**Discussion:**

This study will provide valuable clinical evidence that YSF can help to improve the cognitive function of elderly people with MCI, and the results will be disseminated via conferences and publications.

**Trial registration:**

Chinese Clinical Trial Registry, ChiCTR2000036807. Registered on August 25, 2020.

**Supplementary Information:**

The online version contains supplementary material available at 10.1186/s12906-023-03940-x.

## Background

Chinese people aged 60 and older numbered 264.02 million, or 18.7% of the total population, according to the National Bureau of Statistics of China in the 2020 census. As the Chinese population ages and ageing-associated diseases increase at a rapid rate, the prevalence of dementia among individuals aged 60 years and older was found to be 6.0%, and mild cognitive impairment (MCI) was estimated in 15.5% of individuals of this age group in China [1]. MCI refers to a cognitive decline that does not impair cognitive function significantly, which is generally referred to as a transition state between normal cognition and Alzheimer's disease [2], and compared with normal cognition, MCI is more likely to develop into Alzheimer's disease [3]. Undoubtedly, cognitive impairment is an extremely serious problem, and effective therapy should be administered before the disease worsens. Cholinesterase inhibitors (ChEIs) are commonly used for treating MCI, with very little evidence showing that they can prevent the progression from MCI to dementia. The efficacy of ChEI treatment for MCI remains controversial [4–6]. Thus, novel strategies and prevention methods need to be explored for MCI treatment. We hope that TCM can produce positive effects on clinical treatment, increasing patients’ confidence in the use of Chinese herbs, which can further enhance the efficiency of prevention and treatment.

In recent years, an increasing number of studies have shown that traditional Chinese medicine has a potential multitarget therapeutic effect on early MCI and exerts neuroprotective effects, showing certain application prospects [7–11]. Yi Shen Fang (YSF) granules are traditional Chinese herbal compounds that have been commonly used in MCI treatment in our specialist outpatient clinic. A previous clinical study indicated that YSF could improve cognitive function in elderly patients, especially by improving naming, attention, and delayed recall ability. YSF granules consist of Xiangfu (*Cyperus rotundus*), Rougui (*Cinnamomum verum*), Sharen (*Amomum dealbatum*), Shanzhi (*Gardenia jasminoides*), Shichangpu (*Acorus calamus*), Yuanzhi (*Polygala tenuifolia*), and Xuancaohua (*Hemerocallis lilioasphodelus*).

In previous research on YSF in the treatment of cognitive impairment in vascular dementia rats carried out by our research group, we established rats with two-vessel occlusion and middle cerebral artery occlusion as a vascular cognitive impairment (VCI) rat model [12]. The results of the experiment showed that YSF could inhibit the apoptosis of hippocampal neurons and improve the spatial search ability and localization of VCI rats by regulating the BDNF-TrkB pathway and expression of mTOR protein in the hippocampus [13], which was sponsored by the Natural Science Foundation of Shanghai (No. 17ZR1428100). Based on the above basic research results, we performed a multicentre, double-blind, randomized controlled study of YSF granules combined with acupuncture for elderly adults with MCI to further verify the clinical efficacy on cognitive function. The preliminary results showed that YSF granules combined with acupuncture could improve cognitive function, especially the visuospatial and executive ability of elderly patients with MCI (unpublished data). YSF has been granted the patent of invention by the China National Intellectual Property Administration (Patent No. 201810784587.8) as a TCM composition to improve VCI. Therefore, the aim of this trial is to objectively evaluate the clinical efficacy and safety of YSF in the treatment of elderly patients with MCI based on our previous studies and to further provide a new therapy and clinical basis for the treatment of MCI with TCM.

## Methods

### Study design

This study is a multicentre, randomized, double-blind, parallel-group, controlled trial. The objective of this trial is to evaluate the clinical efficacy and safety of YSF granules in elderly patients with MCI. The trial protocol (version 2.0, April 27, 2020) has been registered with the Chinese clinical trial registry (No. ChiCTR2000036807). The period for research recruitment is from August 5, 2021, to December 31, 2023. We present the following article in accordance with the SPIRIT 2013 reporting checklist. For more details about the checklist, please see Additional file 1.

**Fig. 1:**
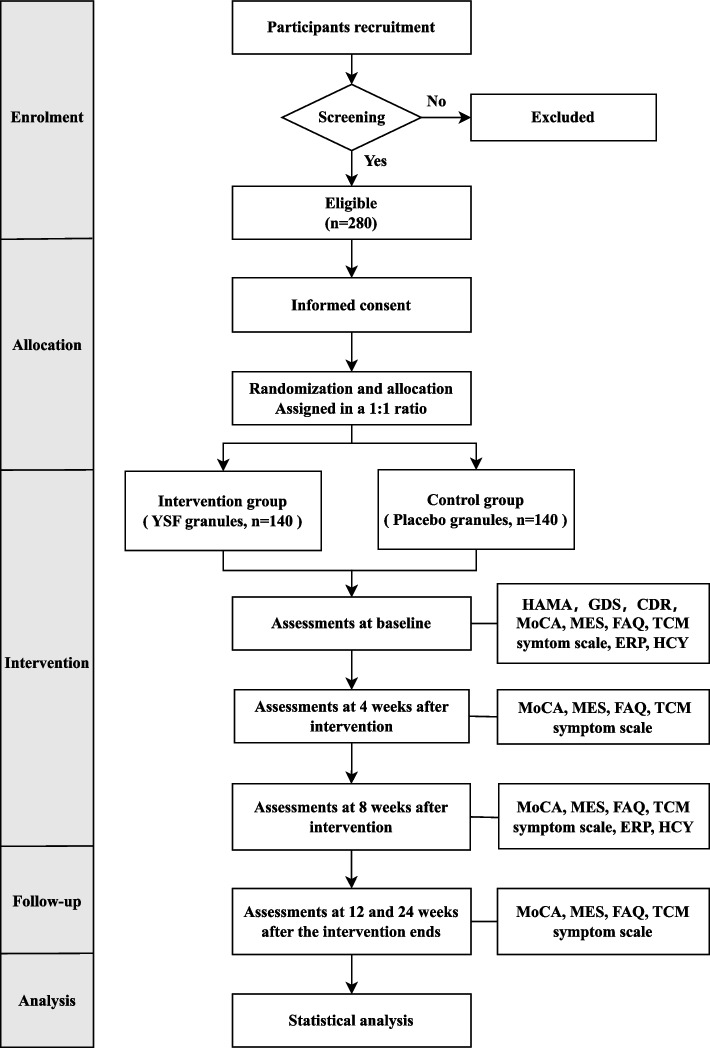
Flowchart of the study trial. YSF: Yi Shen Fang; HAMA: Hamilton Anxiety Scale; GDS: Geriatric Depression Scale; CDR: Clinical Dementia Rating Scale; MoCA: Montreal Cognitive Assessment Score; MES: Memory and Executive Screening; FAQ: Functional Assessment Questionnaire; TCM Symptom Scale: Traditional Chinese Medicine Symptom Scale; ERP: event-related-potentials; HCY: homocysteine

Before the beginning of the clinical study, all researchers will be trained on the standardized process and scale evaluation consistency to ensure the clinical trial quality and data credibility of each centre. A total of 280 elderly patients with MCI will be randomly divided into a treatment group and a control group in a 1:1 allocation ratio. It will be carried out in three clinical centres in Shanghai, 140 subjects will be recruited at Shanghai Municipal Hospital of TCM, and we plan to recruit 70 subjects from Shanghai Qingpu District Hospital of TCM and 70 subjects from Shanghai Jiading District Nanxiang Hospital. All participants will experience a 1-week screening period, with an 8-week treatment period and follow-up visits at 24 and 48 weeks. The primary efficacy outcome measures will be the change in the Montreal Cognitive Assessment (MoCA) and Memory and Executive Screening (MES) scores, which will be used to assess cognitive function following the interventions. The TCM symptom scale is a combined measure of syndrome differentiation and treatment. Safety outcome markers will include hepatic and renal functions, electrocardiogram data, routine blood and urine test results, and severe clinical discomfort. The schedule of enrolment, interventions, assessments and data collection is presented in Table 1. The study will follow the Helsinki Declaration and the Good Clinical Practice guidelines. Ethical approval was granted by the Ethics Committee of Shanghai Municipal Hospital of TCM (approval no. 2020SHL-KY-06). Each subject could only be included in this study once and signed an informed consent form prior to study participation. The results of the study will help provide a clinical basis and TCM treatment plan for standardized diagnosis and treatment of MCI. The flowchart of this study is illustrated in Fig. 1.

**Table 1:** Schedule of enrolment, interventions, and assessments

	STUDY PERIOD					
**STUDY PROCEDURE**	Enrolment	Allocation	Post-allocation		Follow-up	
**TIMEPOINT**	-1-week	0-week	4-week	8-week	12-week	24-week
**ENROLMENT**						
Eligibility screen	×					
Informed consent	×					
Allocation		×				
**INTERVENTIONS**						
Intervention group			×	×		
Control group			×	×		
**ASSESSMENTS**^**a**^						
HAMA	×					
GDS	×					
CDR	×					
MoCA	×	×	×	×	×	×
MES	×	×	×	×	×	×
FAQ	×	×	×	×	×	×
TCM symptom scale	×	×	×	×	×	×
ERP		×		×		
HCY		×		×		
**SAFETY ASSESSMENTS**						
Laboratory tests^b^	×			×		
**Adverse events**			×	×	×	×

^a^Assessments: *HAMA* Hamilton Anxiety Scale, *GDS* Geriatric Depression Scale, *CDR* Clinical Dementia Rating Scale, *MoCA* Montreal Cognitive Assessment Score, *MES* Memory and Executive Screening, *FAQ* Functional Assessment Questionnaire, *TCM Symptom Scale* Traditional Chinese Medicine Symptom Scale, *ERP* event-related-potentials, *HCY* homocysteine

^b^Laboratory tests: routine blood, hepatic (*ALT* alanine aminotransferase, *AST* aspartate aminotransferase) and renal (*BUN* blood urea nitrogen, *Cr* creatinine) functions, urine, faecal and electrocardiogram

### Eligibility criteria

#### Diagnostic criteria

Participants will be diagnosed with MCI according to the 10th International Classification of Diseases (ICD-10) and 2018 Chinese guidelines for the diagnosis and treatment of dementia and cognitive impairment (V): diagnosis and treatment of MCI [14]; the main feature of MCI is the decline in cognitive function, which may include memory impairment and learning or concentration difficulties. Objective tests can often find abnormalities, but the symptoms do not meet the criteria for a diagnosis of dementia, organic amnesia syndrome or paranoia.

#### Inclusion criteria

1. Male and female participants are aged 60 years and older;
2. Clear consciousness, normal hearing and visual abilities are maintained, and individuals have the ability to participate in the neuropsychological activity test;
3. Participants meet diagnostic criteria for MCI in the ICD-10 and should meet the following items at the same time—the patient or participant subjectively feels that there is a decline in cognitive function, impaired cognitive function is noted in objective examination, and daily life function is basically normal;
4. Participants do not meet the diagnostic criteria for dementia—the total score on the Clinical Dementia Rating Scale (CDR), Chinese version, is 0.0 to 0.5;
5. The total score on the MoCA, Beijing version, is below 26 (years of education < 12 years, MoCA score < 25);
6. Participants agree to sign the informed consent form and volunteer for this study.

#### Exclusion criteria

1. Participants with a history of mental disorders such as dissociative disorder, obsessive–compulsive disorder, personality disorder, schizophrenia, bipolar disorder, severe depression disorder, and anxiety disorder will be excluded.
2. Participants with a history of drug or alcohol dependence or drug allergies will be excluded from this study.
3. Participants who are taking antipsychotic medication or medications that affect cognition.
4. Participants with a history of head trauma resulting in loss of consciousness will be excluded.
5. Any participant for whom treatment was accepted in other clinical drug trials before the first study-drug administration and within 4 weeks of the present study will be excluded.
6. Participants with serious gastrointestinal diseases (such as indigestion, gastrointestinal obstruction, or gastroduodenal ulceration) and serious secondary diseases that affect drug absorption will be excluded.
7. Participants with hepatic and renal dysfunction, severe neurological diseases, cardiovascular and cerebrovascular disease complications and a history of other autoimmune diseases and any other contraindication were excluded.
8. Participants with scores on the 30-item version of the Geriatric Depression Scale (GDS-30) higher than 20 will be excluded.
9. Participants with a Hamilton Anxiety Rating Scale (HAMA, 14-item version) score ≥ 21 will be excluded.

#### Termination criteria

1. Patients who experience severe hepatotoxicity and nephrotoxicity related to drugs determined by the researchers during the study.
2. Patients who experience other serious adverse events (AEs).
3. Patients with comorbidities, complications or special physiological changes that may make them unsuitable for further study.
4. Patients who use prohibited prescribed treatment.

#### Randomization

In this study, a stratified block randomization method will be used to divide the subjects in the same centre according to the "centre" first, and then subjects in the same centre will be divided into block randomization groups. This method not only ensures the balance of each known important prognostic factor but also ensures that the number of cases in each treatment group in each centre was close or equal; this method is also convenient for management. To ensure the comparability of the key factors affecting the prognosis of patients in the intervention or control group, the stratified factors considered in this study are centre, sex, age and TCM syndrome patterns. Block randomization requires that the research object of each block be a multiple of the number of groups (twofold or fourfold). The sample size of this experiment is 280 patients, so each group from each area should consist of 4 patients, for a total of 70 blocks. Among them, there were 35 (140/4) blocks from the Shanghai Municipal Hospital of TCM, 18 (70/4) blocks from Shanghai Jiading District Nanxiang Hospital, and 18 (70/4) blocks from the Shanghai Qingpu District Hospital of TCM. To reduce the loss of patients who meet the inclusion and exclusion criteria, the three centres could also set up a small number of blocks for 2 patients (such as fewer elderly patients) according to the actual needs.

At the same time, considering that when the three centres match patients of similar sex, age and TCM syndrome patterns into block groups, it is possible that the number of research objects in a block will be small or could lead to matching difficulties, so each centre should add 6 to 8 random participants (a total of 4 blocks).

#### Blinding method

To control the bias caused by intentional or unintentional subjective factors in the process of clinical trials and the interpretation of results, neither the researchers nor the patients will know whether the test drug or the control drug was used by the patients. Eligible participants will be randomized to the treated group and control group at a 1:1 ratio. Twice unblinding will be used after data collection is completed. The data file will be locked after blinding verification and confirmation, and then the first unblinding will be performed. Data will be clustered to determine whether each number belongs to the A or the B group. The second unblinding will be performed after statistical analysis to determine whether group A or group B corresponds to the treated group or control group.

According to the abovementioned double-blind method, statistical experts will divide the drugs into two groups. Each participant will be assigned a medicine bag that contains all medication to be used during the treatment period. Each centre should distribute the drugs according to the drug number and the order of the subjects.

The research group will prepare an emergency letter for each subject. The envelope will be marked with the subject's drug number, and the inner sealed letter will indicate the subject's group, which will be used for emergency unblinding. The emergency envelopes will be saved at each centre and only opened for a clinical emergency. Once the blinded information is leaked or the opening rate of emergency letters exceeds 20%, the double-blind test is invalid. After sealing, blinded information will be delivered to the sponsor at Shanghai Municipal Hospital of TCM and managed by a specially assigned person.

### Interventions

#### Intervention group

YSF granules (4.24 g/bag, batch number: 201007) are provided by Sichuan Neo-Green Pharmaceutical Technology Development Co., Ltd., which is one of only six TCM formula granule manufacturers approved by the China Food and Drug Administration (Production Licence Number: Chuan 20,160,134). The components of YSF granules are listed in Table 2. Two bag granules will be added to 200 ml of warm water and administered orally every morning and evening after meals for 8 weeks. The granules should be stored at room temperature in a dry and dark location out of reach of children.

**Table 2:** Overview of Yi Shen Fang

	Chinese medicine	Family	Amount (%)	TCMH action
1	Xiangfu (*Cyperus rotundus*)	Cyperaceae	21.13%	Soothe the liver, regulate qi, and regulate menstruation to relieve pain
2	Rougui (*Cinnamomum verum*)	Lauraceae	4.23%	Tonify fire and assist yang, direct fire back to its origin, dissipate cold to relieve pain, and activate blood to unblock the meridians
3	Sharen (*Amomum dealbatum*)	Zingiberaceae	8.45%	Resolve dampness to move qi, warm the middle to inhibit diarrhoea, and prevent miscarriage
4	Shanzhi (*Gardenia jasminoides*)	Rubiaceae	12.68%	Purge fire to except vexed clear heat and drain dampness, cool the blood and detoxify, and disperse swelling and relieve pain
5	Shichangpu (*Acorus calamus*)	Acoraceae	12.68%	Resolve phlegm to open the orifices, resolve dampness and harmonize the stomach, nourish the heart and tonify intelligence
6	Yuanzhi (*Polygala tenuifolia*)	Polygalaceae	12.68%	Tranquillize the patient, tonify intelligence, and resolve phlegm to open the orifices
7	Xuancaohua (*Hemerocallis lilioasphodelus*)	Asphodelaceae	28.17%	Clear heat, drain dampness, and cool the blood to stop bleeding

*TCMH* traditional Chinese medicine herb

#### Control group

The control group will receive granules with a 10% drug concentration (0.42 g/bag) serving as the placebo [15], which the ingredients and the preparation process are the same as the YSF granules (Table 2). Following the principle of a double-blind trial, food colorants and flavouring agents will be added so that the two groups of drugs can be similar in terms of colour, smell, taste and quality. The usage is the same as above.

#### Drug combination

During the study period, no cognitive improvement drugs will be allowed. All combined drugs should be recorded in a case report form (CRF), including the drug name, production batch, usage and dosage, course of treatment, treatment plan and other related information. During the trial, the concurrent use of other TCM preparations will not be allowed. Finally, the researchers will determine whether the participants should withdraw from the study according to the combined drug usage.

### Outcome evaluation

#### Baseline characteristics

Patients’ demographic characteristics including age, gender, ethnicity, marital status, cohabitation status, educational attainment, occupation, predisposing factors, prior therapies, history of illness, medication history, vital signs and weight for height will be considered. Baseline data for the enrolled participants will be recorded before randomization.

#### Primary outcomes

Cognitive status will be evaluated with the MoCA and MES. The primary efficacy endpoints are the improvement rate for MoCA scores and MES scores, which will be measured before and after treatment in both groups. The MoCA has high sensitivity and specificity in distinguishing normal elderly individuals from those with MCI and mild Alzheimer's disease and can detect slight cognitive impairment [16]. The MES has been confirmed to be a valid and easily administered screening tool for MCI, and the score is independent of education and can also be used to evaluate individuals [17].

$$\text{Improvement rate} = \left[\left(\text{posttreatment score}-\text{pretreatment score}\right)/\text{pretreatment score}\right]\times100\%.$$

#### Secondary outcomes

Secondary outcome measures are the FAQ and TCM symptom scale scores before and after intervention. The change in activities of daily living is closely related to the change in cognition and could sensitively reflect the changes in patients' conditions. The FAQ scale involves complex social functions and daily activities and is suitable for the evaluation of patients with mild symptoms. According to the *Guidance Principle of Clinical Study on New Drug of TCM* (2002) [18], we score the change in TCM syndromes of participants before and after intervention.

We will use cognitive event-related potentials (ERPs) as the objective indicator for the assessment of cognitive status. ERP measurement is a sensitive method for detecting cognitive decline, inducing prolonged latency of P300 waves, and has certain specificity for the diagnosis of MCI. Serum homocysteine (HCY) will be detected pre- and posttreatment. Many prospective studies have found that increased t-HCY remained associated with cognitive impairment, which has significant implications for public health [19, 20].

#### Safety evaluation

For the safety evaluation, routine blood, liver function (alanine aminotransferase (ALT) and aspartate aminotransferase (AST)), renal function (blood urea nitrogen (BUN) and creatinine (Cr)), urine, and faecal tests and electrocardiograms will be performed before treatment and after 8 weeks of intervention. Vital signs and all adverse events will be recorded at every study visit during the trial. In case of any adverse event during the study, the clinician in charge of the case shall carefully record the details, including the name and characteristics of the adverse event, occurrence and disappearance time, treatment measures, impact on the primary disease and prognosis, in the CRF.

#### Data management and monitoring

Shanghai Municipal Hospital of Traditional Chinese Medicine will assign an independent quality inspector to oversee the process of the clinical trial. Furthermore, we will strengthen the exchange by telephone or other communication means. The purpose of supervision is as follows: (1) to ensure compliance with the protocol; (2) to ensure the completeness and accuracy of the information entered into the CRF and the dispensed study drug return records; (3) to protect the rights of participants; and (4) to check the accuracy, completeness and legality of the data.

### Statistical plan

#### Sample size calculation

The necessary sample sizes were calculated for the two primary outcomes, namely, the MoCA and MES. With reference to our previous study [21], the following parameters were used: the mean MoCA score (*μ*_r_) in the treatment group was 25.67; the mean MoCA score (*μ*_c_) in the control group was 23.36; we assumed that the superiority margin (Δ) was 1.20; the standard deviation (σ) was 2.21; the type I error rate (α) was set to the usual level, a unilateral value of 0.025), with Z_α/2_ = 1.96, the type II error rate (β) was set to a unilateral value of 0.05, with Z_β_ = 1.645; the ratio (K) of the treatment group size (*n*_1_) to the control group size (*n*_2_) was K = 1 (*n*_1_ = *n*_2_ = 104). Considering that there are three research centres in this study and a 20% dropout rate, the logistic regression analysis suggested that the sample size should be 15 to 30 times the number of indices being analysed plus an extra 30 patients, for a total required sample size of 280 patients (140 patients per group).

For the second primary outcome, we set the mean MES scores to 82.83 in the treatment group and 73.50 in the control group, with an SD of 7.83 [22]. We assumed that the superiority margin was 3.92 (1/2 standard deviation); we calculated that we would need a total of 122 patients (66 per group) to allow for a 20% dropout rate. Of the necessary sample sizes estimated for the two primary outcomes, the larger value was selected as the sample size for this study. The formula for calculating the sample size is as follows:

$$\begin{array}{c}{\mathrm n}_{\mathrm c}=\frac{\left({\mathrm Z}_{\displaystyle\mathrm\alpha/2}\;+\;{\mathrm Z}_{\mathrm\beta}\right)^2\;\mathrm\sigma^2\;\left(1+{\displaystyle\frac1{\mathrm k}}\right)}{\left(\mu_r-\mu_c-\mathit\triangle\right)^2}\\{\mathrm n}_{\mathrm r}={\mathrm{Kn}}_{\mathrm c}\end{array}$$

### Statistical analyses

In this study, the data analysis will be performed on the full analysis set (FAS), per protocol set (PPS) and safety analysis set (SS). The efficacy analysis will be conducted on an intention-to-treat (ITT) basis. The 95% confidence intervals (CIs) will be calculated as exact CIs. For the two primary outcomes, the *P*-value will be adjusted to 0.033. This value was based on a recommendation to divide the pre-specified *P*-value threshold by the value halfway between 1 (representing no adjustment) and the number of primary outcome comparisons (the denominator used for the Bonferroni correction) [23]. All statistical tests are 2-sided, and unless otherwise stated, *P* < 0.05 will be considered statistically significant. When the primary outcome measures are missing, the researchers will combine statistical analysis and clinical experience to judge whether to exclude the subject or replace the missing data with the last observation carried forward (LOCF).

The measurement data will be subject to normal distribution and homogeneity of variance, which is expressed as the mean ± standard deviation. Comparisons between groups will be performed using an independent 2-sample t test. Comparisons between multiple time points will be analysed using repeated-measures ANOVA, multivariate ANOVA or paired t test. When the data do not meet the criteria of normality and homogeneity, the results will be expressed as the median. The Wilcoxon rank sum test will be used to compare differences between groups. Before and after treatment, numerical variables and ordered categorical variables measured over multiple time points will be analysed using generalized estimating equations and a mixed effects model. The enumeration data will be expressed as frequency (n), constituent ratio (%) or ratio (%). If the variable data type is unordered, categorical comparisons between groups will be performed by the χ^2^ test. The ordered categorical variable data will be analysed using the rank sum test. Comparisons of two-way ordered data with different properties within contingency tables will be made with the linear chi-square test or rank sum test. Multivariate data analysis will be conducted using multiple linear regression, the K-M test, Cox regression or a trend test. All statistical analyses will be performed with IBM SPSS Statistics 24.0.

## Discussion

Population ageing is an inevitable trend for the world’s population [24], and China has one of the world’s fastest-growing demographics of older individuals worldwide [25]. It is estimated that by mid-century, the elderly population in China will reach 400 million, accounting for approximately 30% of the total population; accordingly, the prevalence of cognitive impairment in elderly individuals will increase. Shanghai was the first city to become a superaged society, in which ageing individuals constitute the highest percentage of the city’s population. The office of the Shanghai Working Committee on Ageing and the Municipal Bureau of Statistics recently released the latest data and showed that on December 31, 2019, there were 5.1812 million older adults aged 60 years and older, accounting for 35.2% of the total population registered in Shanghai, and 2729 older people were over 100 years old in Shanghai. In China, the population ageing problem has increased the number of individuals with incapacitating diseases, accounting for 3% of the total number of elderly people, which has led to substantial personal and social burdens [26]. Therefore, how can health problems brought about by population ageing be proactively addressed? Efficacious therapy and prevention strategies are urgently needed to cope with age-related neurodegenerative diseases, including cognitive impairment and dementia, which are difficult problems that we need to overcome in medical and health research [27].

Cognitive function involves various aspects of working memory, execution function, reasoning ability, verbal ability, and ability to perform activities of daily living [28]. Cognitive impairment refers to the impairment of one or more functions in the above areas, which can affect the social function and quality of life of patients to varying degrees and even lead to death in severe cases. MCI is a transitional state between normal ageing and dementia [29]. Cognitive impairment is not only a simple medical problem but also a serious social problem. To the best of our knowledge, the incidence rate of MCI is 6.7% at 60–64 years of age, and the prevalence rate is 10 to 20% in the elderly population over 65 years old and 25.2% in the population aged 80–84 years old [30]. More than half of MCI patients will progress to dementia within 5 years, and only a small number of MCI patients can maintain stable cognitive function or even return to normal [31]. Approximately 10% of MCI patients convert to Alzheimer's disease (AD) every year, and the incidence of dementia in MCI is 10 times higher than that in healthy elderly people [32]. Therefore, early intervention for MCI is very important for delaying the occurrence and development of dementia, improving the ability to perform activities of daily living, prolonging the survival period and reducing the burden of care and care; additionally, the early stage of MCI is considered to be the best intervention period to delay or reverse cognitive impairment.

Chinese herbal formulas have been widely used to improve cognitive function with a good curative effect. In the last few years, many investigators have engaged in research on TCM interventions in cognitive disorders to improve the management of MCI by TCM and to take the disease stage within the Alzheimer disease continuum seriously. TCM shows good clinical efficacy and characteristic advantages of early TCM intervention in cognitive impairment to provide an accurate basis to examine effective TCM therapies to delay the progression of cognitive impairment.

### Limitations

The following issues are considered limitations of the study. First, the 8-week treatment cycle in this study is relatively short. Cognitive impairment may require long-term maintenance medication to delay the development of the disease to prevent progression to dementia. Second, biomarkers and neuroimaging characteristics associated with MCI in the early symptomatic period are not yet clear. The changes in ERP and serum HCY may not fully reflect the curative effect of TCM on MCI. Subjective measurements combined with imaging may need to be performed to find the correlation between early brain tissue changes and MCI in further studies. Third, participants in this trial have fewer comorbidities. MCI is often comorbid with cardiovascular and cerebrovascular diseases, neurodegenerative or affective disturbance [33, 34], but the trial is aimed at increasing the pertinence of inclusion criteria and standardizing the drug to the disease, so it lacks certain generalizability. Finally, the 6-month follow-up period is rather short. MCI is a chronic, progressive disease that requires long-term follow-up to assess cognitive changes. Therefore, we may not observe the key node of cognitive impairment, further development, or transformation after the termination of treatment.

Bearing these limitations in mind, we will conduct long-term follow-up to record the prognosis of MCI patients treated with Chinese herbs. There is a dire need to strengthen health care systems with additional medical institutions and place more emphasis on the health of elderly people to improve the quality of life of these individuals. We are calling for more effective ways of delaying the progress of cognitive impairment as well as improve the prognosis.

### Trial status

The trial began recruitment in August 2021 and participant recruitment has been delayed because of the COVID-19, which is still in progress.

## Supplementary Information

**Additional file 1.** SPIRIT 2013 Checklist.

## Data Availability

Not applicable.

## Abbreviations

HAMA: Hamilton Anxiety Scale
GDS: Geriatric Depression Scale
CDR: Clinical Dementia Rating Scale
MoCA: Montreal Cognitive Assessment Score
MES: Memory and Executive Screening
FAQ: Functional Assessment Questionnaire
TCM Symptom Scale: Traditional Chinese Medicine Symptom Scale
ERP: Event-related-potentials
HCY: Homocysteine
ALT: Alanine aminotransferase
AST: Aspartate aminotransferase
BUN: Blood urea nitrogen
Cr: Creatinine
TCMH: Traditional Chinese medicine herb
